# Positive and Negative Risk-Taking in Adolescence and Early Adulthood: A Citizen Science Study During the COVID-19 Pandemic

**DOI:** 10.3389/fpsyg.2022.885692

**Published:** 2022-06-06

**Authors:** Lysanne W. te Brinke, Renske van der Cruijsen, Kayla H. Green, Eveline A. Crone

**Affiliations:** ^1^Department of Psychology, Education and Child Studies, Erasmus School of Social and Behavioral Sciences, Erasmus University Rotterdam, Rotterdam, Netherlands; ^2^Faculty of Social and Behavioural Sciences, Leiden University, Leiden, Netherlands

**Keywords:** sensation seeking, risk-taking, COVID-19, prosocial behavior, contributing to society, life satisfaction, citizen science, participatory research

## Abstract

Sensation seeking is an important underlying factor of both positive and negative forms of risk-taking during adolescence and early adulthood. However, macro-factors such as the global COVID-19 pandemic may influence sensation seeking opportunities and risk-taking behaviors that are considered negative and positive. Therefore, the primary aim of this study was to examine the associations between sensation seeking and behaviors that are considered positive or negative forms of risk-taking during the Covid-19 pandemic in a sample of adolescents and early adults (*N* = 660, *M*_age_ = 22.91, *SD* = 3.14). Using citizen science methods, negative risk-taking was defined as taking unaccepted risks, such as falsifying vaccination reports or deliberately contracting COVID-19. Positive risk-taking was defined as taking socially accepted risks, such as balancing between the risk to infect elderly people and the need to socialize with peers. Results showed that participants with higher levels of sensation seeking took more positive and negative COVID-19 related risks. An additional finding was that sensation seeking was positively associated with the need to contribute to society. This indicates that during adolescence and early adulthood, sensation seeking may be a driving factor for both positive (i.e., socially accepted) and negative (i.e., socially unaccepted) risk-taking in the context of a high-stake global pandemic, arguing against a one-direction negative relation between sensation seeking and risk-taking.

## Introduction

The developmental period of adolescence is characterized as a time of heightened sensation seeking (Steinberg et al., 2018). Adolescents frequently desire to seek novel, thrilling, and intense situations or experiences. This drive for sensations is associated with behaviors that potentially have detrimental consequences for the health of self and others, such as reckless driving and having unprotected sex (Hansen and Breivik, 2001; Zuckerman, 2007). However, sensation seeking may also underlie behaviors that potentially have positive consequences for self and others, such as taking a risk to help others (i.e., prosocial risk-taking; Do et al., 2017; Blankenstein et al., 2020). In addition, sensation seeking is related to personality factors such as extraversion and psychoticism (Glicksohn and Zuckerman, 2013; Zuckerman and Glicksohn, 2016). During the global COVID-19 crisis, adolescents’ opportunities to seek novel situations have been diminished due to lockdowns, stay-at-home advice, and social distancing. This societal challenge has also considerably changed what has traditionally been interpreted as “negative” or “positive” risk-taking, given that socializing and being in close contact with others can be interpreted as both societally acceptable and a risk to the health of self and others. Therefore, the main goal of this study is to examine associations between sensation seeking and behaviors that are considered negative or positive forms of risk-taking during the global COVID-19 crisis.

Negative risk-taking is generally defined as risky behavior that may have adverse consequences for the health self and others (Moore and Gullone, 1996). This form of risky behavior is typically viewed as socially unacceptable or antisocial, such as reckless driving, having unprotected sex, and drinking alcohol (Fryt et al., 2021). According to the dual systems model of adolescent risk-taking, heightened vulnerability to negative risk-taking in adolescence may be a consequence of the combination of relatively high inclinations to seek exciting, novel experiences and relatively immature capacities for self-control (Steinberg, 2010; Steinberg et al., 2018). Reward-sensitivity on the one hand and impulsivity/self-control on the other hand are expected to develop at different time scales (Steinberg, 2010). Thus, according to theory, the desire to seek novel, thrilling, and intense situations or experiences (i.e., sensation seeking) is positively linked to negative risk-taking, especially for adolescents with low self-control abilities. Empirical research confirms this positive association between sensation seeking and various forms of negative risk-taking during adolescence and early adulthood (e.g., Lauriola et al., 2014). In early adolescence, increases in sensation seeking and increases in the inclination to take risks are associated with and predictive of engagement in risky behaviors such as alcohol use (MacPherson et al., 2010). Sensation seeking is also found to predict both occasional and frequent negative risk-taking (e.g., reckless behavior on the road) among high school students (Desrichard and Denarié, 2005). Moreover, antisocial adolescents are found to score above the normative mean of impulsiveness and venturesomeness, two dimensions that are linked to sensation seeking (Lipperman-Kreda and Glicksohn, 2010). Antisocial risk-taking adults are also found to score higher on sensation seeking than prosocial risk-takers, risky sport engagers and individuals who do not engage in risky activities (Gomà-i-Freixanet, 1995, 2001). Lastly, early adults with higher scores on sensation seeking measures tend to engage in riskier financial behaviors (Worthy et al., 2010). Thus, sensation seeking can be considered an important determinant of various forms of negative, socially unaccepted forms of risk-taking.

Over the past decade, it has become evident that sensation seeking tendencies may also underlie positive forms of risk-taking. Positive risk-taking is generally defined as risky behavior that may have beneficial consequences for the well-being of self and others (Duell and Steinberg, 2019). This form of risky behavior is typically viewed as socially acceptable or prosocial, such as speaking up for someone in a challenging social situation or helping someone who is socially excluded (Vrijhof et al., 2016). During early and middle adolescence, sensation seeking is associated with greater positive risk-taking (Hansen and Breivik, 2001; Duell and Steinberg, 2020). In a sample of 10–18-year-old adolescents, sensation seeking was, for example, positively associated with instrumental risk-taking (e.g., “to achieve something in life one has to take risk”; Siraj et al., 2021). Moreover, adolescents and adults (15–70 +) who were willing to engage in risky sports – which can be considered a form of positive risk-taking – were found to have relatively high sensation seeking scores (Breivik et al., 2017). Adult alpinists, mountain climbers, and mountain skiers also score higher on sensation seeking than adults who do not engage in these types of risky sports (Gomà-i-Freixanet, 1991). Furthermore, adults who work in risky professions (i.e., “prosocial risk-takers” such as bomb-disposal experts, anti-terror operatives, bodyguards) score relatively high on sensation seeking (especially the thrill and adventure seeking dimension; Glicksohn and Bozna, 2000; Glicksohn and Rechtman, 2011). Thus, sensation seeking can also be considered an important determinant of various positive forms of risk-taking.

Recent studies have moved beyond examining positive and negative risk-taking as distinct constructs (Duell and Steinberg, 2020; Fryt and Szczygiel, 2021). It is proposed that positive and negative risk-taking are correlated, and share the same underlying factors such as sensation seeking (Duell and Steinberg, 2019). Although the magnitude of these associations may depend on the context and type of negative and positive risk-taking behavior that is assessed, research shows that general negative risk-taking (e.g., drinking alcohol, having unprotected sex) and general positive risk-taking (e.g., trying new foods, sharing something personal) are positively correlated with a small to medium effect size (*r* = 0.31 in Duell and Steinberg, 2020; *r* = 0.20 in Fischer and Smith, 2004). This means that adolescents who frequently engage in positive forms of risk-taking, may also show more negative forms of risk-taking (Duell and Steinberg, 2019). In addition, studies that examined the shared association between sensation seeking and negative/positive forms of risk-taking, show that these associations are of comparable magnitude (Fischer and Smith, 2004; Patterson et al., 2019; Duell and Steinberg, 2020). Thus, sensation seeking plays a central role in the development of both positive and negative forms of risk-taking during adolescence and early adulthood.

Despite the shift toward a more integrated study of negative and positive forms of risk-taking, relatively little is known about the role that macro-factors such as societal context, culture, and socioeconomic status play in risk-taking tendences (for a recent study on socioeconomic factors, see Jia et al., 2021). This integrated macro-factor perspective is important, because risk-taking preferences are found to be domain- and context-specific (Frey et al., 2017; Zhang and Palma, 2022). This means that positive and negative risk-taking in one domain (i.e., positive social risks during COVID-19) does not necessarily correlate with risk-taking in another domain (i.e., negative health risks during COVID-19). Moreover, these macro-factors may influence which risk-taking behaviors are considered “negative” or “positive,” and consequently, how these behaviors relate to sensation seeking tendencies. In prior research, the classification of negative (i.e., socially unacceptable and posing a threat to self or others) and positive (i.e., socially acceptable and beneficial to self and others) risk-taking, has been made within a research context (e.g., Duell and Steinberg, 2020). Although helpful, this distinction fails to take the teen culture and context into account, whereas it is known that adolescents differ from adults in how “risky” they perceive specific behaviors (Cohn et al., 1995). Thus, it may very well be that behaviors that adults (e.g., researchers) perceive as socially unacceptable, are considered socially acceptable within the teen culture. The current study aims to overcome these barriers, by defining risk-taking within the context of teen culture. To this end, a novel citizen science approach is used, in which adolescents/young adults from the target population actively engaged in multiple phases of the research process (Cuevas-Parra, 2020). This enabled us to examine risk-taking aspects that are defined as socially acceptable or unacceptable by adolescents and early adults themselves.

A second important contextual factor that needs to be taken into account is the global Covid-19 pandemic. In order to decrease the spread of the COVID-19 virus, rules and regulations such as stay-at-home advice, lockdowns, and social distancing have been imposed. Up until now, little is known about the association between adolescents’ sensation seeking tendencies and positive versus negative risk-taking during the COVID-19 pandemic. A study examining sex differences in rule-breaking behavior during COVID-19 among adults, found, however, that sensation seeking was positively related to COVID-19 related rule-breaking behaviors (i.e., leaving home for non-essential reasons), especially among males (DeGrace et al., 2021). Research also showed that adults who are prone to experience boredom – a factor linked to sensation seeking (Dahlen et al., 2004) - were more likely to break rules of social isolation (Boylan et al., 2021). Based on these results, we expected that sensation seeking would predict COVID-19 related negative and positive risk-taking behaviors among adolescents and early adults.

Besides the importance of incorporating macro-factors into the study of positive and negative risk-taking, there is also a need to examine if – and how – risk-taking during adolescence and early adulthood relates to positive developmental outcomes, such as becoming a contributing member to society and experiencing life satisfaction. Zooming in on these associations is especially relevant during the COVID-19 crisis since this societal challenge has diminished the life satisfaction of adolescents all over the globe (e.g., Magson et al., 2021). Moreover, adolescence is not only characterized as a period of heightened sensation seeking and risk vulnerability (Steinberg, 2004; Steinberg et al., 2018), but also as a period in which individuals have a high need to contribute to society (Fuligni, 2019; Crone and Fuligni, 2020). Contributing to society is linked to civic engagement (i.e., prosocial and political contributions to community and society; Wray-Lake et al., 2019). We define societal contribution as an intra-societal form of prosocial behavior, that consists of behaviors that are beneficial to society, such as raising one’s voice to injustice, volunteering for community service, and supporting family or friends (i.e., informal care). Research during the initial phase of the COVID-19 crisis shows that the need of adolescents for societal contribution did not change in comparison to pre-pandemic levels (van de Groep et al., 2020), but this may have changed with the longer duration of the pandemic. Thus, little is known about the associations between risk-taking and societal contribution during COVID-19. Therefore, a secondary goal of this study is to explore whether sensation seeking and risk-taking are associated with adolescents’ and early adults’ societal contributions during COVID-19. Due to the novelty of the societal context, no specific hypotheses were formulated and the associations between sensation seeking, risk-taking and societal contribution/life satisfaction were tested exploratively.

To summarize, the primary aim of this study is to examine the associations between sensation seeking and behaviors that adolescents consider positive or negative forms of risk-taking during the COVID-19 pandemic. We hypothesize that sensation seeking is related to both positive and negative forms of risk-taking, and that these associations are stronger for adolescents as compared to early adults, given the heightened levels of sensation seeking and risk-taking in mid-to-late adolescence (Steinberg et al., 2008; Duell et al., 2018). The secondary aim of this study is to examine whether sensation seeking and risk-taking during the COVID-19 pandemic are associated with positive developmental outcomes (i.e., societal contributions and life satisfaction).

## Materials and Methods

### Participants

In total, 660 adolescent and young adults between 16 and 30 years-old participated in this study (*M*_age_ = 22.91, *SD* = 3.14)^1^. Of the participants, 68.8% identified as female and 29.3% as male. The ethnicity of 90.5% of the participants was Dutch. Of the participants that did not identify themselves as of Dutch origin, 14.3% identified as North-European (e.g., German), 12.7% identified as Surinamese-Dutch, 11.1% as Chinese-Dutch, 9.5% as Turkish-Dutch, 9.5% as Indonesian-Dutch, 7.9% as Moroccan-Dutch, 7.9 as South-European, 4.8% as Latin-American, and 3.2% as Antillean-Dutch. With regard to education levels, 3.9% followed secondary education, 2.6% intermediate vocational education, 15.2% higher professional education, 59.3% university education, and 19.0% was graduated.

### Measures

#### Sensation Seeking

General sensation seeking was measured with the Brief Sensation-Seeking Scale (Hoyle et al., 2002; Pechorro et al., 2018). This scale consists of 8 items (e.g., “I would love to have new and exciting experiences, even if they are illegal”). The scale includes the four dimensions of sensation seeking that are identified by Zuckerman (1971); experience seeking (i.e., desire to seek experience for its own sake), boredom susceptibility (i.e., dislike of repetition of experience, being restlessness when things are unchanging), thrill and adventure seeking (i.e., desire to engage in sports or other activities involving elements of speed and danger), and disinhibition (i.e., loss of social inhibition). Responses were given on a 5-point scale ranging from 1 (*strongly disagree*) to 5 (*strongly agree*). The scale was translated from English to Dutch by the first and second author, back translated by a research assistant, and checked by the citizen scientists for clarity. This procedure and the final translated version of the questionnaire is displayed in the Supplementary Material 1. Cronbach’s alpha was 0.82, McDonald’s omega was 0.82.

#### Primary Outcomes

##### Negative Risk-Taking During COVID-19

Negative risk-taking during COVID-19 was operationalized as taking risks that could harm the health of self and others. Specifically, three items were included that measured whether the adolescents would be inclined to create more freedom for themselves during COVID-19 restrictions by falsifying polymerase chain reaction-tests (pcr-tests), falsifying vaccination status reports, and deliberately risking contracting COVID-19 (e.g., “would you try to create more freedom for yourself with the corona passport by falsifying a negative test”). Responses were given on a 5-point scale ranging from 1 (*no, not at all*) to 5 (*yes, definitely*). Cronbach’s alpha was 0.75, McDonald’s omega was 0.78.

##### Positive Risk-Taking During COVID-19

Positive risk-taking was operationalized as balancing between the risk to infect elderly people (i.e., adhering to social distancing from elderly and restricting contact with elderly) and the need to socialize with peers (i.e., adhering to social distancing from peers and restricting contact with peers), while considering the general expected vaccination rate in the population. The positive risk-taking measure included a 12-item questionnaire that was created for this study. Specifically, six items measured willingness to social distance and restrict contact with *elderly* (e.g., “If 30% of the people in the Netherlands are vaccinated, to what extent do you think that you: keep your distance from elderly people”), and six items measured willingness to social distance and restricting contact with *peers* (e.g., If 60% of the people in the Netherlands are vaccinated, to what extent do you think you will: see your peers more”). Both for elderly and peers, questions were asked for 30, 60, and 90% vaccination rates. Responses were given on a 5-point scale ranging from 1 (*I would definitely not do that*) to 5 (*I would definitely do that*). Social-distancing scores were recoded, to ensure that a higher score was indicative of higher non-adherence to safety measures. Mean scores were created for both elderly and peers. Cronbach’s alpha was 0.79 for elderly and 0.80 for peers. McDonald’s omega was 0.79 for elderly and 0.81 for peers.

Based on this questionnaire, an interaction variable was created to capture positive risk-taking. This risk-taking variable indicated that higher socially accepted risk-takers during COVID-19: (a) differentiate more between *non-adherence toward elderly versus peers* (i.e., higher non-adherence toward peers versus elderly reflects the balance between the need to socialize with peers and the risk of elderly people to become seriously ill when contracting COVID-19), and (b) differentiate more between *low versus high vaccination rates* (i.e., non-adherence is riskier with low vaccination rates in the general population). First, the mean difference between non-adherence to peers versus elderly was calculated (mean peers – mean elderly). Second, the mean increase in non-adherence from 30 to 90% vaccination rates was calculated (mean 90% – mean 30%). Third, the mean difference was multiplied by the mean increase^2^, which resulted in a positive risk-taking variable that ranged from –2.08 to 4.88. A calculation example for a high and low positive risk-taking participant is provided in the Supplementary Material 2.

#### Secondary Outcomes

##### Societal Contribution

Societal contribution was measured with five items from the adapted General Contribution to Society questionnaire (GCS; van de Groep et al., 2020). Specifically, two items measured the need for societal contribution during COVID-19 (e.g., “I would like to raise my voice during this crisis”), and three items measured the perceived opportunities for societal contribution (e.g., “I feel that I am valuable to society during this crisis”). Responses were given on a 7-point scale ranging from 1 (*Totally not agree)* to 7 (*Totally agree).* Cronbach’s alpha was 0.40 and McDonald’s omega was 0.41. Moreover, the correlation between needs and commitments appeared too small (*r* = 0.16). Therefore, the scales were included separately in the analyses.

##### Life Satisfaction

Life satisfaction was measured with a single item on a 10-point scale (e.g., “What grade would you give your life right now on a scale of 1 to 10”).

### Procedure

**FIGURE 1:**
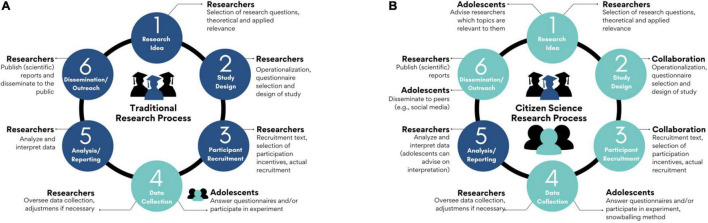
Graphical display of a possible traditional **(A)** research process, following the empirical cycle, and a citizen science process **(B)**, with the target group being actively engaged in multiple phases of the research process.

We collected data in April–May 2021, using a novel citizen science approach (Cuevas-Parra, 2020). A critical element of the citizen science approach is that adolescents from the target group have an active role in the study. Thus, in contrast to a traditional approach in which the target group (i.e., adolescents and early adults) are passively involved in the data collection phase of a study (Figure 1A), the current study actively involved the target group in multiple phases of this study (Figure 1B).

#### Research Idea, Study Design, and Recruitment Phase

In the first phases of this study, three young adults from the target population (22–24 years-old) were actively involved as citizen scientists. They advised the researchers on topics that were important for adolescents and young adults during the COVID-19 crisis. From initial discussions between the citizen scientists and researchers, it became apparent that within the teen culture, non-adherence to COVID-19 was not necessarily a “rebellious” or “negative” act, but rather, a way to support each other – and themselves – in the need to socialize with friends. This inspired the researchers to examine non-adherence to COVID-19 regulations as a form of positive risk-taking.

In the study design phase, the three citizen scientists and researchers collaborated in the operationalization and questionnaire development through online meetings and online chat contact. The first goal of this collaboration was to operationalize negative and positive risk-taking within the context of teen culture. According to the citizen scientists, negative forms of risk-taking were not uncommon among adolescents and young adults and included deliberately contracting COVID-19 or falsifying test results, in order to generate more freedom to themselves. The citizen scientists agreed that these behaviors were unacceptable (i.e., negative) forms of risk-taking within the teen culture, because these behaviors had negative health consequences for self and others. With regard to positive risk-taking, it was of critical importance to the citizen scientist to make a distinction between non-adherence to COVID-19 measures toward peers versus elderly. The citizen scientist agreed that differentiation between non-adherence to peers and elderly (i.e., higher non-adherence toward peers, especially if risks were lower due to high vaccination rates in the general population) were considered positive (i.e., socially accepted) forms of risk-taking within the teen culture, because these behaviors would have positive consequences for the mental health of self and others.

After the citizen scientists agreed on the operationalization of risk-taking, the questionnaires were developed collaboratively. The citizen scientist provided feedback on all measures and checked the general sensation seeking measure for meaning and clarity (see Supplementary Material 2). Subsequently, a recruitment text was written by the citizen scientists (see Supplementary Material 3). In the recruitment text, they highlighted that the collaborative goal of the study was to make sure that in future decisions regarding COVID-19 measures, the opinion of adolescents and young adults was taken into account, and that participants could help by filling out the questionnaires and forwarding the recruitment text to their friends. This recruitment text was posted by the citizen scientists and researchers on several social media websites, and the citizen scientists circulated the text in several WhatsApp groups for adolescents and young adults.

#### Data Collection Phase

A “snowballing” sampling approach was used (Baltar and Brunet, 2012), which allowed not only the three citizen scientist, but all participants, to be actively involved in the data collection phase of this study. This resulted in a cumulative increase of participants, with the majority of participants responding to the questionnaires within 10 days (see Supplementary Material 3). Adolescents and young adults who wished to participate in the study signed informed consent and subsequently filled-out the questionnaires via Qualtrics software (Qualtrics, Provo, UT, United States). After finishing the survey, participants could indicate whether they wished to join a “raffle” for AirPods (a suggestion of the citizen scientists). No other participation incentives where included. The study was reviewed and approved by the Ethics Committee of the Erasmus School of Social and Behavioral Sciences of Erasmus University Rotterdam.

#### Analysis and Dissemination Phase

The citizen scientists were not actively involved in the data analysis/interpretation phase. After the study was finished, the descriptive statistics were, however, summarized by the researchers. Together with the citizen scientist, these findings were communicated via social media platforms (i.e., outreach). This allowed us to share the most important descriptive findings with the target population. Moreover, a Dutch policy report was written (see te Brinke et al., 2021). The Dutch National Institute for Public Health and the Environment (RIVM) used the findings from this report in briefings to the government and COVID-19 strategy boards, to ensure that the opinion of adolescents and young adults was considered in future decisions regarding COVID-19 measures.

### Analyses Plan

Data was analyzed with SPSS Statistics v. 28.0 (IBM Corp, 2020). First, we examined whether it was necessary to control for gender and age effects, by testing the main effects of age (regression analysis) and gender (ANOVA) on primary (sensation seeking and risk-taking) and secondary (societal contribution and life satisfaction) variables^3^. To avoid alpha error inflation caused by multiple testing, *p*-values were corrected with the Holm–Bonferroni correction method (Holm, 1979; Gaetano, 2013).

### Primary Analyses

To examine associations between sensation seeking and risk-taking, stepwise regression analyses were performed. Age and gender were entered as covariates in step 1, and sensation seeking in step 2. We also tested to which extent sensation seeking was a *specific* predictor of each outcome variable, by adding positive/negative risk-taking as covariates to the regression model. Subsequently, we examined whether age moderated the associations between sensation seeking and risk-taking with moderation analyses. For these analyses, we used the PROCESS SPSS macro version 4.0 (Hayes, 2017). Sensation seeking was included as predictor variable, age as moderator, and gender as covariate.

### Secondary Analyses

To examine whether negative/positive risk-taking and sensation seeking predicted societal contribution, another set of stepwise regression analyses was performed. Needs and opportunities for societal contribution were included as dependent variables. Age and gender were entered as predictors in step 1 (to control for the main effects), negative/positive risk-taking in step 2, and sensation seeking in step 3. Subsequently, to examine whether age moderated the associations between sensation seeking and societal contribution, moderation analyses were performed, using the PROCESS SPSS macro version 4.0 (Hayes, 2017). Sensation seeking was included as predictor variable, age as moderator, and gender as covariate.

To examine whether risk-taking, societal contribution, and sensation seeking predicted life satisfaction, another stepwise regression analysis was performed. Life satisfaction was included as dependent variable. Age and gender were entered as predictors in step 1, negative/positive risk-taking in step 2, societal need/contribution in step 3, and sensation seeking in step 4.

Lastly, we exploratively looked at the four dimensions of sensation seeking separately, as predictors of risk-taking, societal contribution, and life satisfaction. The results of these *post hoc* analyses are reported in the Supplementary Material 4.

### Missing Data

Missing data ranged from 0.2 to 7.0% (see Table 1). Attrition analyses showed that participants with missing data on any of the study variables (*N* = 52) did not differ from participants with complete data (*N* = 608), regarding gender (*X*^2^ = 1.38, *p* = 0.240), ethnicity (*X*^2^ = 0.01, *p* = 0.986), or age [*F*(1,658) = 2.59, *p* = 0.108]. Moreover, Little’s MCAR test indicated that the data was missing completely at random (*X*^2^ = 25.71, *p* = 0.423). Therefore, listwise deletion was used as missing data handling strategy.

**TABLE 1:** Number of participants, descriptive statistics, and correlations among study variables.

	*N*	*M*	*SD*	1	2	3	4	5	6	7
(1) Age	660	22.91	3.14	–						
(2) Gender^a^	651	0.70	0.46	0.08*	–					
(3) Sensation seeking	614	3.36	0.85	−0.16**	−0.09*	–				
(4) Negative risk-taking	628	1.31	0.70	–0.02	–0.07	0.20**	–			
(5) Positive risk-taking	659	1.46	1.04	−0.20**	–0.01	0.20**	–0.04	–		
(6) Societal needs	617	5.02	1.14	–0.08	0.10*	0.20**	0.06	0.06	–	
(7) Societal opportunities	617	4.09	1.22	0.04	0.09*	0.00	−0.13**	0.01	0.16**	–
(8) Life satisfaction	613	6.05	1.60	0.11**	–0.00	−0.20**	−0.17**	–0.07	–0.03	0.23**

*^a^Gender is coded as 0 (boy) 1 (girl).*

*Indicates p < 0.05; **indicates p < 0.01.

## Results

### Descriptive Statistics

**FIGURE 2:**
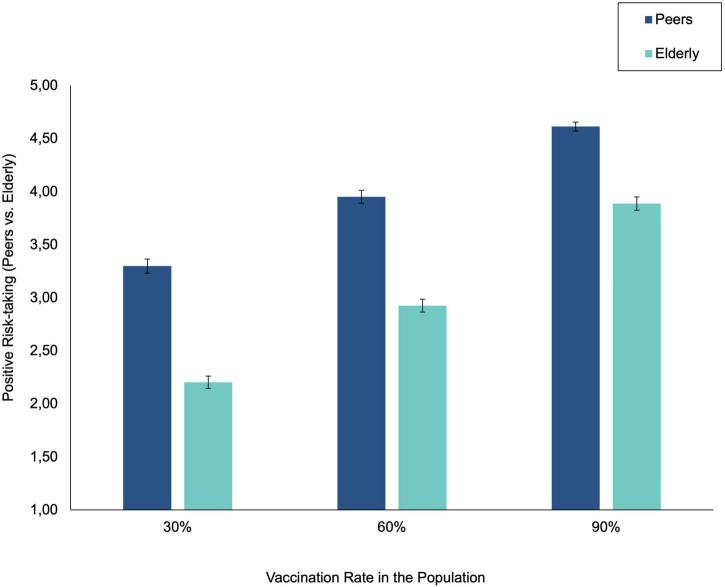
Average positive risk-taking to peers versus elderly (i.e., non-adherence to social distancing and contract restriction). Larger differences between elderly and peers, combined with lower distinctions between high versus low vaccination rates, are indicative of positive risk-taking during COVID-19.

Descriptive statistics and correlations among study variables are displayed in Table 1. Negative risk-taking and positive risk-taking were not correlated. In total, 72.5% of the participants indicated that they were not willing to falsify pcr-tests, vaccination status reports or deliberately risk contracting COVID-19. Thus, the degree of negative risk-taking was relatively low (see Table 2). The mean scores of non-adherence to social distance and restricting contact measures toward elderly versus peers are displayed in Figure 2. In total, 89.2% of the participants differentiated at least one scale point (on a 5-point scale) in their average difference in non-adherence toward elderly in contrast to peers, and the average vaccination-related increase in non-adherence was 1.60 scale points. This indicates that the majority of participants balanced between the risk to infect elderly people and the need to socialize with peers. Thus, the degree of positive risk-taking during COVID-19 was relatively high. Regarding societal contribution, the descriptive statistics (Table 2) show that the reported need for societal contribution is significantly higher than the experienced opportunity (*t* = 15.09, *p* < 0.001).

**TABLE 2:** Coefficients for stepwise regression analyses with age, gender (step 1), and sensation seeking (step 2) as predictors of negative risk-taking and positive risk-taking.

	Negative risk-taking^b^					Positive risk-taking^c^				
	*b*	*SE*	β	*p*	*p’*	*b*	*SE*	β	*p*	*p’*
Gender^a^	–0.07	0.06	–0.05	0.267	0.534	0.08	0.09	0.03	0.393	0.393
Age	0.00	0.01	–0.00	0.990	0.990	–0.06	0.01	–0.18	<0.001	<0.001
Sensation seeking	0.16	0.03	0.19	<0.001	<0.001	0.21	0.05	0.17	<0.001	<0.001

*p’ is the Holm–Bonferroni adjusted p-value.*

*^a^Gender is coded as 0 (boy) 1 (girl).*

*Change statistics of adding sensation seeking when age and gender are already in the regression:*

*^b^ΔR^2^ = 0.04, ΔF(1,601) = 22.84, p < 0.001; ^c^ΔR^2^ = 0.03, ΔF(1,601) = 17.70, p < 0.001.*

### Associations Between Sensation Seeking and Risk-Taking During COVID-19

#### Gender and Age Effects on Sensation Seeking and Risk-Taking

ANOVAs showed that boys (*M* = 3.47, *SD* = 0.79) reported significantly higher levels of sensation seeking than girls [*M* = 3.31, *SD* = 0.87; *F*(1,603) = 4.84, *p* = 0.028, η^2^ = 0.01]. No gender differences in negative risk-taking [*F*(1,617) = 2.99, *p* = 0.084, η^2^ = 0.01] or positive risk-taking [*F*(1,648) = 0.02, *p* = 0.886, η^2^ < 0.00] were found.

**FIGURE 3:**
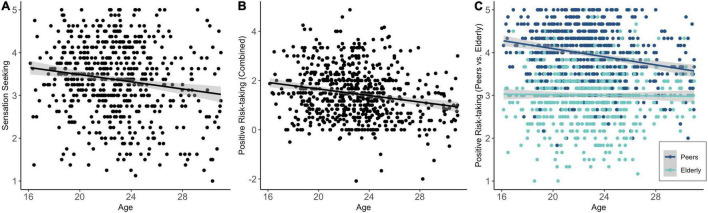
Linear age effects on sensation seeking **(A)**, general positive risk-taking **(B)**, and positive risk-taking to peers versus elderly (i.e., non-adherence to social distancing and contract restriction), with larger differences between peers and elderly being indicative of higher positive risk-taking **(C)**.

Regression analyses showed that age predicted a significant amount of variance in sensation seeking [*R*^2^ = 0.02, *df*(1,612) = 15.10, *p* < 0.001]. As displayed in Figure 3A, older adolescents reported lower levels of sensation seeking (*b* = –0.04, *SE* = 0.01, β = –0.16, *p* < 0.001). Age predicted also a significant amount of variance in positive risk-taking [*R*^2^ = 0.04, *df*(1,657) = 27.54, *p* < 0.001]. Specifically, older participants reported lower levels of positive risk-taking (*b* = –0.07, *SE* = 0.01, β = –0.20, *p* < 0.001; Figure 3B). Follow-up analyses showed that the association between age and positive risk-taking could be explained by a heightened need of younger adolescents to socialize with peers (Figure 3C). Specifically, there was a significant negative association between age and non-adherence of COVID-19 measures toward peers [*R*^2^ = 0.05, *df*(1,658) = 31.20, *p* < 0.001; *b* = –0.05, *SE* = 0.01, β = –0.21, *p* < 0.001], but age did not predict a significant amount of variance in non-adherence of COVID-19 measures toward elderly [*R*^2^ = 0.00, *df*(1,657) = 0.17, *p* = 0.678]. No age effect on negative COVID-19 related risk-taking was found [*R*^2^ = 0.00, *df*(1,626) = 0.21, *p* = 0.651].

#### Sensation Seeking Effects on Risk-Taking

Regression analyses showed that sensation seeking was a significant predictor of negative risk-taking (Table 2). When adding positive risk-taking behavior to the model, the association between sensation seeking and negative risk-taking remained significant (*b* = 0.17, *SE* = 0.03, β = 0.21, *p* < 0.001, adjusted *p* = 0.024). Thus, participants with higher levels of sensation seeking took more negative COVID-19 related risks (i.e., they were more inclined to falsify test results or contract COVID-19 deliberately).

**FIGURE 4:**
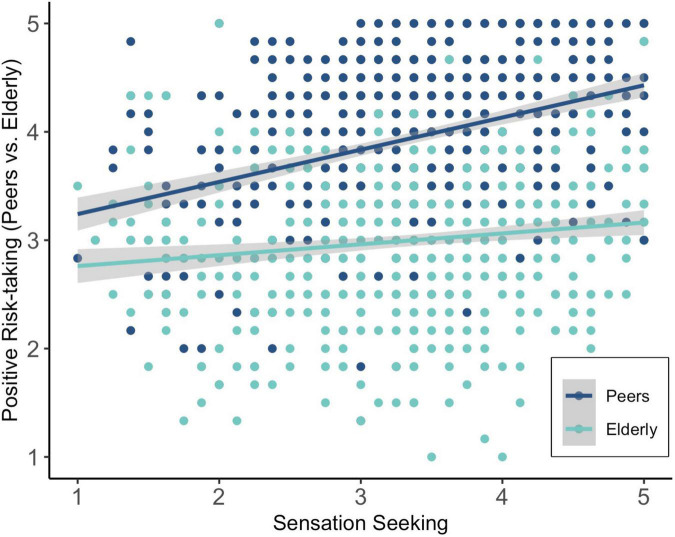
Association between sensation seeking and positive risk-taking to peers versus elderly (i.e., non-adherence to social distancing and contract restriction), with larger differences between peers and elderly being indicative of positive risk-taking.

Sensation seeking was also a significant predictor of positive risk-taking (Table 2). When adding negative risk-taking to the model, the association between sensation seeking and positive risk-taking remained significant (*b* = 0.23, *SE* = 0.05, β = 0.18, *p* < 0.001, adjusted *p* < 0.001). This indicates that participants with higher levels of sensation seeking took more positive COVID-19 related risks. Follow-up analyses showed that the association between sensation seeking and positive risk-taking could be explained by non-adherence toward both peers (*b* = 0.27, *SE* = 0.03, β = 0.33, *p* < 0.001, adjusted *p* = 0.009) and elderly (*b* = 0.10, *SE* = 0.03, β = 0.12, *p* = 0.003, adjusted *p* = 0.009). These associations are displayed in Figure 4. Thus, participants with higher levels of sensation seeking took more positive risks during COVID-19 (i.e., larger differences between non-adherence to social distancing in relation to peers versus elderly people, and high versus low vaccination rates).

#### Moderation by Age

Moderation analyses showed that both for negative risk-taking (*b* = 0.01, *SE* = 0.01, *p* = 0.436, adjusted *p* > 0.999) and positive risk-taking (*b* = 0.02, *SE* = 0.02, *p* = 0.282, adjusted *p* = 0.845), the moderation effect was not significant. Thus, the association between sensation seeking and risk-taking during COVID-19 was not moderate by age.

### Associations Between Sensation Seeking, Risk-Taking, and Societal Contribution During COVID-19

#### Gender and Age Effects on Societal Contribution

Girls (*M* = 5.09, *SD* = 1.10) scored significantly higher than boys (*M* = 4.85, *SD* = 1.22) on the need to contribute to society (*F* = 6.14, *p* = 0.013, η^2^ = 0.01) during the COVID-19 crisis. Girls (*M* = 4.17, *SD* = 1.20) also experienced more opportunities for societal contribution than boys (*M* = 3.94, *SD* = 1.23; *F* = 4.83, *p* = 0.028, η^2^ = 0.01). No gender differences in life satisfaction were found (*F* = 0.01, *p* = 0.973, η^2^ = 0.00).

**FIGURE 5:**
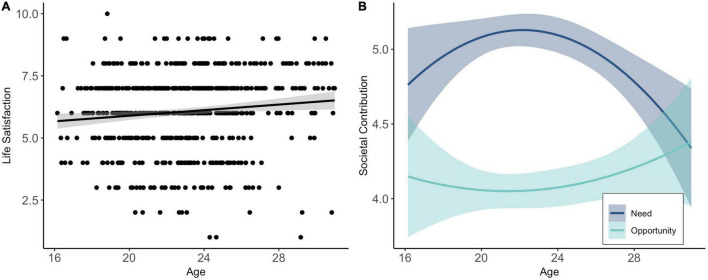
Linear age effects on life satisfaction during COVID-19 **(A)** and quadratic age effects on need and opportunities for societal contribution during COVID-19 **(B)**.

Regression analyses showed that age predicted a significant amount of variance in life satisfaction [*R*^2^ = 0.11, *df*(1,611) = 7.20, *p* = 0.007]. Older participants reported higher levels of life satisfaction during COVID-19 (*b* = 0.06, *SE* = 0.02, β = 0.11, *p* = 0.007; Figure 5A). No significant age effects on the need to [*R*^2^ = 0.08, *df*(1,615) = 3.82, *p* = 0.051] or experienced opportunities for [*R*^2^ = 0.04, *df*(1,615) = 1.00, *p* = 0.317] societal contributions were found. Exploratory follow-up analyses with quadratic age effects, showed, however, that the need for societal contribution [*R*^2^ = 0.02, *df*(1,614) = 6.02, *p* = 0.003] could best be explained by a quadratic age effect (linear age: *b* = 0.45, *SE* = 0.17, β = 1.23, *p* = 0.008; quadratic age: *b* = –0.01, *SE* = 0.001, β = –1.31, *p* = 0.004). As displayed in Figure 5B the need to contribute to society during COVID-19 peaked in late adolescence, around age 23.

#### Sensation Seeking and Risk-Taking Effects on Societal Contribution

**FIGURE 6:**
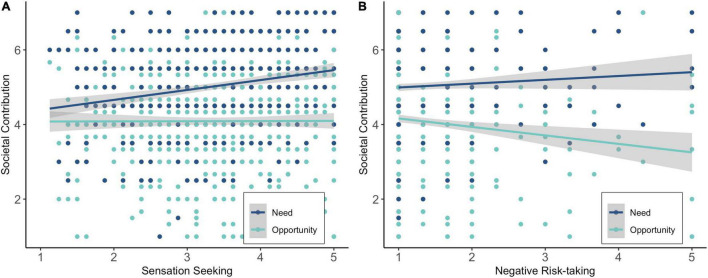
Sensation seeking **(A)** and negative risk-taking **(B)** as predictors of the need to and opportunities of societal contribution during COVID-19.

Regression analyses showed that sensation seeking was a significant positive predictor of the need for societal contribution, whereas both negative and positive risk-taking variables were not (Table 3). This indicates that participants with higher levels of sensation seeking experienced a stronger need to contribute to society during COVID-19 (i.e., speak up for change, help society members), see Figure 6A.

**TABLE 3:** Coefficients for stepwise regression analyses with age, gender (step 1), risk-taking (step 2) and sensation seeking (step 3) as predictors of societal needs and opportunities.

	Societal needs^b^					Societal opportunities^c^				
	*B*	*SE*	β	*p*	*p’*	*b*	*SE*	β	*p*	*p’*
Gender^a^	0.30	0.10	0.12	0.002	0.008	0.20	0.11	0.08	0.057	0.228
Age	–0.02	0.02	–0.05	0.200	0.600	0.01	0.02	0.04	0.369	0.738
Negative risk-taking	0.07	0.07	0.04	0.320	0.640	–0.24	0.07	–0.14	<0.001	0.004
Positive risk-taking	0.02	0.05	0.05	0.587	0.640	0.02	0.05	0.02	0.711	0.738
Sensation seeking	0.25	0.06	0.19	<0.001	<0.001	0.08	0.06	0.05	0.217	0.651

*p’ is the Holm–Bonferroni adjusted p-value.*

*^a^Gender is coded as 0 (boy) 1 (girl).*

*Change statistics of adding sensation seeking when age and gender are already in the regression:*

*^b^ΔR^2^ = 0.04, ΔF(1,601) = 22.84, p < 0.001; ^c^ΔR^2^ = 0.03, ΔF(1,601) = 17.70, p < 0.001.*

Sensation seeking and positive risk-taking did not significantly predict perceived opportunities for societal contribution (Table 3). Negative risk-taking was, however, a significant negative predictor of perceived opportunities for societal contribution (Table 3). This indicates that participants who took more negative COVID-19 related risks perceived fewer opportunities to contribute to society (i.e., they felt less valuable to society during the COVID-19 crisis), see Figure 6B.

#### Moderation by Age

Both for societal needs (*b* = –0.02, *SE* = 0.02, *p* = 0.355, adjusted *p* = 0.710) and perceived societal opportunities (*b* = 0.01, *SE* = 0.02, *p* = 0.802, adjusted *p* > 0.999), the moderation effect was not significant. Thus, the association between sensation seeking and societal contribution was not moderate by age.

### Sensation Seeking, Risk-Taking, and Societal Contribution as Predictors of Life Satisfaction

**FIGURE 7:**
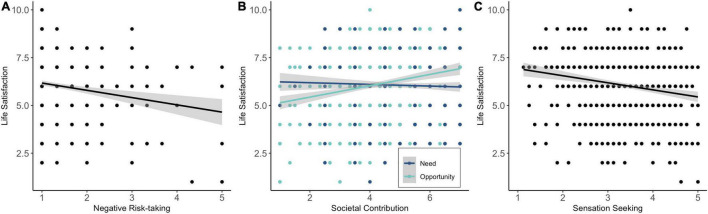
Negative risk-taking **(A)**, societal contribution **(B)**, and sensation seeking **(C)** as predictors of life satisfaction during COVID-19.

The results of regression analyses with life satisfaction as dependent variable can be found in Table 4, and significant associations are displayed in Figure 7. Negative risk-taking and sensation seeking were significant negative predictors of life-satisfaction. Thus, participants with higher sensation seeking needs and higher willingness to take negative COVID-19 related risks, reported lower levels of life satisfaction (see Figures 7A,B). In contrast, perceived opportunities for societal contribution were a positive predictor of life satisfaction. Thus, participants who felt more valuable to society during the COVID-19 crisis, experienced higher levels of life satisfaction (see Figure 7C). Positive risk-taking and the reported need for societal contribution did not significantly predict life satisfaction.

**TABLE 4:** Coefficients for stepwise regression analysis with age, gender (step 1), negative/positive risk-taking (step 2), societal needs/opportunities (step 3) and sensation seeking (step 4) as predictors of life satisfaction.

	*b*	*SE*	β	*p*	*p’*
Gender^a^	–0.15	0.14	–0.04	0.284	0.852
Age	0.04	0.02	0.07	0.077	0.308
Negative risk-taking	–0.28	0.09	–0.12	0.003	0.015
Positive risk-taking	–0.06	0.06	–0.04	0.378	0.852
Societal needs	–0.00	0.06	–0.00	0.968	0.968
Societal opportunities	0.27	0.05	0.20	<0.001	0.025
Sensation seeking	–0.29	0.08	–0.15	<0.001	0.001

*p’ is the Holm–Bonferroni adjusted p-value.*

*^a^Gender is coded as 0 (boy) 1 (girl).*

*Change statistics of adding risk-taking (step 2), societal needs/opportunities (step 3), and sensation seeking (step 4) when age and gender are already in the regression: step 2: ΔR^2^ = 0.03, ΔF(2,594) = 10.63, p < 0.001; step 3: ΔR^2^ = 0.04, ΔF(2,592) = 12.39, p < 0.001; step 4: ΔR^2^ = 0.02, ΔF(1,591) = 13.88, p < 0.001.*

## Discussion

The primary aim of this study was to examine associations between sensation seeking and behaviors that adolescents consider socially acceptable or unacceptable forms of risk-taking during the COVID-19 pandemic. Using citizen science methods, we found that within the teen culture, behaviors that threaten the health of self and others, such as falsifying test results or deliberately contracting COVID-19, were considered negative (i.e., socially unaccepted) forms of risk-taking. In contrast, behaviors that enabled adolescents to socialize with peers despite COVID-19 measures, were considered positive (i.e., socially accepted) forms of risk-taking, because these behaviors may strengthen the (mental) health of self and others. High positive risk-taking was conceptualized by the citizen scientists as differentiating in non-adherence of social distancing measures toward peers versus elderly, while at the same time taking the general vaccination status in the population into account.

The first question we addressed, was whether sensation seeking relates to both negative and positive forms of COVID-19 related risk-taking. As expected, our results showed that adolescents and early adults with higher levels of sensation seeking took more positive and negative COVID-19 related risks. These findings are consistent with prior studies showing that sensation seeking is associated with general positive and negative risk-taking, such as trying new foods, sharing something personal, drinking alcohol, and having unprotected sex (Fischer and Smith, 2004; Patterson et al., 2019; Duell and Steinberg, 2020). Moreover, these findings are consistent with prior studies showing that sensation seeking is associated with context-specific (i.e., job-related) positive and negative risk-taking in adulthood (Glicksohn and Bozna, 2000; Glicksohn and Rechtman, 2011). The current study thus builds on existing literature, by showing that during adolescence and early adulthood, sensation seeking is not only associated with general risk-taking, but also with context-specific forms of risk-taking. This indicates that sensation seeking may be a driving factor for both positive and negative risk-taking in the context of a high-stake global pandemic.

In line with previous research, the current study also found main effects of age and gender on sensation seeking and risk-taking. In our sample of 16- to 30-year-olds, younger adolescents were found to display higher levels of both sensation seeking and positive risk-taking. The association between age and positive risk-taking could for a large part be explained by a heightened need of younger adolescents to socialize with their peers despite Covid-19 regulations. These findings are in line with previous research showing that sensation seeking peaks in mid-to-late adolescence (Steinberg et al., 2008) and steadily declines thereafter (Roth et al., 2007). Previous research also shows that adolescents are more concerned for the social risk of being excluded than (early) adults (Andrews et al., 2020). Thus, it is possible that the non-adherence to COVID-19 regulations toward peers during mid-adolescence, can in part be explained by a risk of social exclusion. Moreover, the current study found that boys reported higher levels of sensation seeking than girls. This finding is in line with previous research showing that both during adolescence and adulthood, overall levels of sensation seeking are higher for boys than girls (e.g., Roth et al., 2007; Cross et al., 2013). It should be noted that the current study found these gender differences by using the Brief Sensation-Seeking Scale, whereas the developers of these scale (Hoyle et al., 2002) reported that gender effects were absent with this scale – a characteristic that they interpreted as a performance improvement of this measure. The current study shows that also when using the brief sensation-seeking scale, researchers should take potential gender differences into account.

The second question we addressed, was whether sensation seeking and risk-taking were associated with positive developmental outcomes during the COVID-19 pandemic (i.e., societal contribution and life satisfaction). With regard to sensation seeking, we found a significant positive association with the experienced need for societal contribution, and a negative association with life satisfaction. Thus, participants with higher levels of sensation seeking experienced a stronger need to contribute to society during COVID-19 (i.e., speak up for change, help society members), but at the same time, sensation seeking was also linked to lower life satisfaction during the COVID-19 pandemic. With regard to risk-taking, we found that participants who displayed a higher willingness to take negative COVID-19 related risks perceived fewer opportunities for societal contributions (i.e., they felt less valuable to society during the COVID-19 crisis) and lower levels of life satisfaction. In contrast, participants who felt more valuable to society during the COVID-19 crisis, experienced higher levels of life satisfaction. These findings are in line with prior research showing that higher levels of fun seeking (e.g., trying something new when expecting it to be fun) are related to higher mean levels of prosocial behavior (Blankenstein et al., 2020), and that higher levels of negative risk-taking (e.g., fighting) are related to lower levels of emotional wellbeing (Bergman and Scott, 2001).

The unique contribution of sensation seeking to both negative and positive risk-taking, and to both positive and negative outcomes, suggests that during the developmental period of adolescence and early adulthood, sensation seeking may be a differential susceptibility marker (Ellis et al., 2011). Specifically, high sensation seeking may be related to positive outcomes (i.e., positive risk-taking, the need to contribute to society) in some environments whereas in other environments, high levels of sensation seeking may be related to negative outcomes (i.e., negative risk-taking and low life-satisfaction). The current study did not directly take environmental influences into account, but it is possible that exposure to stressors, such as household chaos and financial difficulties, may have sent some sensation seeking participants up on a path of negative COVID-19 related outcomes. Research shows that adolescents who were more exposed to stressors (i.e., inequality of online homeschooling, family stress) experienced higher instability of negative emotions during the COVID-19 crisis (Green et al., 2021). Moreover, adolescents from low SES backgrounds display higher levels of general negative risk-taking, such as riding a bike after drinking alcohol (Jia et al., 2021). The interpretation that sensation seeking may be a differential susceptibility marker, is in line with the suggestion from Blankenstein et al. (2020) with regard to “fun seeking.” Specifically, fun seeking was found to contribute to both rebelliousness and inter-personal prosocial behavior (Blankenstein et al., 2020). Future research may examine the possible role of sensation seeking domains (i.e., disinhibition versus thrill and adventure seeking; Glicksohn et al., 2018) as a differential susceptibility marker, by following the development of sensation seeking, risk-taking, and intra-personal/intra-societal risk-taking over time in a diverse sample of adolescents and early adults.

The finding that sensation seeking was negatively related to life satisfaction during COVID-19, may be considered unexpected, as previous research has shown that university students with high sensation seeking tendencies are more satisfied in their daily life, especially on days in which they experienced physical pleasure (Oishi et al., 2001). Moreover, sensation seeking is found to be positively associated with subjective wellbeing in an adult sample, and this association was stronger for younger adults as compared to older adults (Ravert and Donnellan, 2021). Similar positive associations between sensation seeking and life satisfaction are found during adolescence (Çelik, 2015). These studies were, however, all conducted before the COVID-19 crisis. It may be that the negative impact of the COVID-19 pandemic on life-satisfaction is more severe for adolescents and early adults who have high sensation seeking tendencies, due to a diminished possibility to seek novel and thrilling experiences during the pandemic. Studies conducted during the COVID-19 pandemic show that among adults, sensation seeking is positively correlated with the perceived impact of the COVID-19 pandemic on social life and free time activities (Gröndal et al., 2021). Moreover, boredom proneness of adults (i.e., a propensity toward experiencing boredom), is found to mediate the association between perceived COVID-19 related stress and emotional distress (Yan et al., 2021).

Lastly, the current study also showed that the need to contribute to society during COVID-19 peaked in (late) adolescence. Moreover, the experienced need for societal contribution was significantly higher than the perceived opportunities. This indicates that there may be a disbalance between the need that adolescents have to raise their voice and help community members, and the degree to which they feel that they can actually contribute. To our knowledge, this is the first study that differentiated between experienced needs and opportunities for societal contribution. Our findings are, however, consistent with the theoretical assumption of Fuligni (2019) that the developmental phase of adolescence is characterized by an increased need to contribute. Moreover, Fuligni (2019) argues that although community programs offer adolescents opportunities to make meaningful contributions, these programs remain out of reach for the majority of adolescents due to lacking resources (e.g., time, personnel). Future research is needed to examine whether the disbalance between the needs and opportunities for societal contribution can be replicated to contexts outside of the COVID-19 situation.

### Strengths, Limitations, and Future Directions

Strengths of the current study include the use of citizen science methods, which enabled us to define context-specific positive and negative risk-taking within the teen culture. In addition, this method enabled us to check whether the items of the translated version of the sensation seeking measure that we used, also represented sensation seeking aspects to the target population. Moreover, the active involvement of adolescents and early adults in the data collection phase of this study (i.e., through snowballing methods), enabled participants to not only be passive participants, but also active contributors to this study. At the same time, the use of snowballing methods can also be considered a limitation, because we had little control in the recruitment phase. As such, some groups of adolescents (i.e., girls and individuals with relatively high educational levels) may have been overrepresented, which may limit the generalizability of our findings.

A second limitation was that the scale that was used to measure negative risk-taking consisted of only three items. Although the internal consistency of the scale appeared to be good, this may have limited our ability to examine the full range of potential negative COVID-19 related risks-taking behaviors. A potentially related limitation was that, in contrast to earlier studies (Fischer and Smith, 2004; Duell and Steinberg, 2020), negative and positive risk-taking were not correlated. Thus, adolescents and early adults who took more negative COVID-19 related risks did not necessarily take more positive COVID-19 related risks. A possible explanation for this finding may be that the frequency of negative risk-taking was relatively low, whereas the frequency of positive risk-taking was relatively high. As a result, the shared variance between the situation specific risk-taking domains may have been limited.

Other limitations include the cross-sectional nature of this study and the use of only self-report measures. Lastly, the current study did not include the full adolescent period, since the youngest participants were 16 years old. This limitation may explain why we did not find an age-moderation effect on the association between sensation seeking and COVID-19 related risk-taking. Since both sensation seeking and risk-taking are heightened in mid-to-late adolescence (Steinberg et al., 2008; Duell et al., 2018), we expected that the association between sensation seeking and risk-taking was stronger for adolescents as compared to early adults. Indeed, in a prior longitudinal study, the risk between sensation seeking and negative risk-taking (i.e., smoking) was found to be strongest in mid-adolescence (i.e., between 12 and 16), and became non-significant in young adulthood (i.e., from age 20 onward; Lydon-Staley and Geier, 2018). Moreover, a meta-analysis that examined the association between sensation seeking and negative risk-taking as measured with the Balloon Analog Risk Task showed that sensation seeking was more likely to influence risk-taking during both middle–late adolescence and young adulthood than during early adolescence (Lauriola et al., 2014). Thus, there may be sensitive windows in development during which relations between sensation seeking and risk-taking are stronger, and these windows may differ depending on the type of risk-taking. Future research may consider to take a wider age group into account, for example from early adolescence into young adulthood, and use longitudinal measures to examine the sequential effects within individuals.

## Conclusion and Implications

This study showed that sensation seeking in adolescence and young adulthood is associated with both negative and positive behaviors during the COVID-19 crisis. On the one hand, young people who have higher sensation seeking tendencies display higher negative risk-taking during COVID-19, for example through falsifying test results or deliberately contracting COVID-19 – behaviors that threaten the health of self and others. Higher levels of sensation seeking were also linked to lower experienced life satisfaction during COVID-19. On the other hand, high levels of sensation seeking were associated with higher levels of positive risk-taking. Differential non-adherence of social distancing measures toward peers versus elderly, may be a way for young people with sensation seeking tendencies to act on their need to socialize with peers. Higher levels of sensation seeking were also linked to higher needs to contribute to society. Theoretically, these findings show that sensation seeking may be a differential susceptibility marker that relates to both positive and negative domain- and context-specific risk-taking.

At the same time, this study shows that providing young people with possibilities to contribute may be an important future direction for both science and society. Within the COVID-19 crisis, the findings of this study may implicate that the experienced life satisfaction of young people can be promoted by offering them more opportunities to make valuable contributions to society, for example by upscaling community programs or by creating possibilities for discussions with policy makers. These opportunities to contribute might be especially needed for adolescents who experience high levels of sensation seeking tendencies. Offering possibilities to contribute might make adolescents and young adults feel more engaged with and valuable to society (Fuligni, 2019; Pontes et al., 2019). At the same time, this may also lead to policy and practices that better take the needs of young people into account (Yeager et al., 2018), which may not only be relevant during the COVID-19 crisis, but also in future societal challenges that the current generation of adolescents and early adults will have to face.

## Data Availability Statement

The original contributions presented in the study are publicly available. This data can be found here: doi: 10.25397/eur.19786453.

## Ethics Statement

The studies involving human participants were reviewed and approved by the Ethics Committee of the Erasmus School of Social and Behavioral Sciences of Erasmus University Rotterdam. The participants provided their written informed consent to participate in this study.

## Author Contributions

LB, RC, and KG collected the data. LB analyzed the data and drafted the manuscript. RC, KG, and EC revised the manuscript. All authors have read and approved the final submitted version of the manuscript.

## Conflict of Interest

The authors declare that the research was conducted in the absence of any commercial or financial relationships that could be construed as a potential conflict of interest.

## Publisher’s Note

All claims expressed in this article are solely those of the authors and do not necessarily represent those of their affiliated organizations, or those of the publisher, the editors and the reviewers. Any product that may be evaluated in this article, or claim that may be made by its manufacturer, is not guaranteed or endorsed by the publisher.

## Footnotes

1 This study was part of a larger citizen science project. The goal of this project was to examine the needs of young people in the “way out” of the COVID-19 crisis. In an earlier publication (in Dutch), we reported descriptive statistics from all participants, aged 16–39 (te Brinke et al., 2021). In the current study, only data from participants aged 16–30 is taken into account due to the focus on adolescence and early adulthood. Moreover, data from *N* = 161 participants could not be included, because these participants had missing data on all study variables. Participants with missing data on all study variables were significantly younger (*M*_age_ = 22.31, *SD* = 2.97, *F* = 4.01, *p* = 0.027).

2 To prevent that participants with negative mean difference scores (mean peers < mean elderly) and negative mean increases (mean 30% > mean 90%) would receive positive risk-taking scores, negative mean increase scores were set to 1.00.

3 Age and gender were included as potential covariates because it has been reported in the literature that adolescents score higher on sensation/seeking and risk-taking than (early) adults (e.g., Mahalik et al., 2013; Steinberg et al., 2018). Moreover, higher levels of sensation-seeking and risk-taking are reported for males compared to females (e.g., Zuckerman et al., 1978; Mahalik et al., 2013), although Hoyle et al. (2002) report the absence of gender effects on sensation seeking as measured with the Brief Sensation-Seeking Scale.
