# Supplementary material for: Positive and Negative Risk-Taking in Adolescence and Early Adulthood: A Citizen Science Study During the COVID-19 Pandemic
Source: Front Psychol. 2022 Jun 6;13:885692. doi: 10.3389/fpsyg.2022.885692 (PMC9207949; doi:10.3389/fpsyg.2022.885692)
Supplement: Supplementary file 1 [file Data_Sheet_1.PDF]

Supplementary Material S1
The Brief Sensation-Seeking Scale: Dutch Translation and Checks for Clarity

In this supplementary document, we explain the procedure of *translating* the Brief Sensation-Seeking Scale in Dutch and *checking* with citizen scientists whether the items of this translated version are indeed considered indicative of sensation seeking for adolescents and young adults. The starting point was the original version of the Brief Sensation-Seeking Scale (Table A) in English (Hoyle et al., 2002; Maloney et al., 2012).

First, two researchers (LB and RC) translated the original version into Dutch. Any discrepancies were discussed, which resulted in the first translated version of the questionnaire (Table B).

Table A. Original version of the Brief Sensation-Seeking Scale (from Hoyle et al., 2002)

**Experience seeking**
1. I would like to explore strange places
5. I would like to take off on a trip with no preplanned routes or timetables
**Boredom susceptibility**
2. I get restless when I spend too much time at home.
6. I prefer friends who are excitingly unpredictable.
**Thrill and adventure seeking**
3. I like to do frightening things.
7. I would like to try bungee jumping.
**Disinhibition**
4. I like wild parties.
8. I would love to have new and exciting experiences, even if they are illegal.

Responses were indicated on five-point scales labeled, ''strongly disagree'', ''disagree'', ''neither disagree nor agree'', ''agree'', and ''strongly agree''

Table B. First translated version of the Brief Sensation-Seeking Scale

**Experience seeking**
1. Ik heb de behoefte om onbekende plekken te verkennen
5. Ik ga graag op reis zonder vooraf geplande routes of planning
**Boredom susceptibility**
2. Ik word onrustig als ik te veel tijd thuis doorbreng
6. Ik heb het liefst vrienden die lekker spontaan en onvoorspelbaar zijn
**Thrill and adventure seeking**
3. Ik vind het leuk om spannende en/of enge dingen te doen*
7. Ik zou graag een keer willen bungeejumpen
**Disinhibition**
4. Ik hou van feesten die uit de hand lopen*
8. Ik zou het leuk vinden om nieuwe en spannende ervaringen te hebben, ook al zijn ze illegaal

** Items were changed in final version based on feedback from citizen scientists, see Table D*

Subsequently, we provided the citizen scientists with a definition of sensation seeking, and asked them to check the translated version of the questionnaire for: a) meaning (does this item represent ‘sensation seeking’ to you, b) clarity (is this translated item clear to you). With regard to meaning, the citizen scientist indicated that all items represented various degrees of sensation seeking to them.

However, they identified two items (item 3 and item 4) as unclear. They suggested to delete the and/or statement in item 3, which resulted in “Ik vind het leuk om enge dingen te doen" (which can be translated to “I like doing frightening things”). With regard to item 4 (“I like wild parties”), they felt that the original Dutch translation “Ik hou van feesten die uit de hand lopen” (which can be translated to “I like parties that run out of control”) was a bit too much sensation seeking (e.g., “this feels like a party that the police has to end”). They indicated that most of their (sensation seeking) peers would not recognize themselves in this item. The citizen scientists and researchers discussed possible alternative translations, and the citizen scientists indicated that “feestjes” (Dutch for small parties) would be more inclusive then “feesten’ (Dutch for big parties). This resulted in “Ik hou van heftige feestjes” (“I like wild parties”).

Finally, the adjusted version was backtranslated into English (Table C). Even though there were some minor formulation differences compared to the original version (Table A), the meaning of the translated items did not differ from their original mean.

Table C. Backtranslation of the Dutch Brief Sensation-Seeking Scale

**Experience seeking**
1. I have the desire to explore unknown places
5. I like to travel without pre-planned routes or itineraries
**Boredom susceptibility**
2. I get restless when I spend too much time at home
6. I prefer to have friends who are spontaneous and unpredictable
**Thrill and adventure seeking**
3. I like doing scary things
7. I would like to go bungee jumping one day
**Disinhibition**
4. I like wild parties
8. I would like to have new and exciting experiences, even if they are illegal

The final version of the Dutch Brief Sensation-Seeking scale, along with the item statistics and internal reliability, is displayed in Table D. The internal reliability of the total scale was good (0.82, Table D), and comparable to the English version (0.76; Hoyle et al., 2002), and the Portuguese version (.82 for males, .85 for females; Pechorro et al., 2018).

Table D. Final version of the Dutch Brief Sensation-Seeking Scale and Descriptive Statistics* (te Brinke, van der Cruijsen, Green, & Crone, 2022)

	M	*SD*
**Experience seeking**		
1. Ik heb de behoefte om onbekende plekken te verkennen	4.13	1.01
5. Ik ga graag op reis zonder vooraf geplande routes of planning	3.39	1.34
**Boredom susceptibility**		
2. Ik word onrustig als ik te veel tijd thuis doorbreng	3.72	1.27
6. Ik heb het liefst vrienden die lekker spontaan en onvoorspelbaar zijn	3.68	1.11
**Thrill and adventure seeking**		
3. Ik vind het leuk om enge dingen te doen	3.06	1.23
7. Ik zou graag een keer willen bungeejumpen	2.99	1.58
**Disinhibition**		
4. Ik hou van heftige feestjes	3.28	1.37
8. Ik zou het leuk vinden om nieuwe en spannende ervaringen te hebben, ook al zijn ze illegaal	2.66	1.27
Total (Cronbach's alpha = .82)	3.36	0.85

Responses were indicated on five-point scales: ''helemaal mee oneens, ''mee oneens'', ''niet eens, niet oneens'', ''mee eens'', and ''helemaal mee eens''

** N = 660, M_{age} = 22.91, SD = 3.14*

References

Hoyle, R. H., Stephenson, M. T., Palmgreen, P., Lorch, E. P., & Donohew, R. L. (2002). Reliability and validity of a brief measure of sensation seeking. *Personality and Individual Differences*, *32*(3), 401–414. https://doi.org/10.1016/S0191-8869(01)00032-0

Maloney, P. W., Grawitch, M. J., & Barber, L. K. (2012). The multi-factor structure of the Brief Self-Control Scale: Discriminant validity of restraint and impulsivity. *Journal of Research in Personality*, *46*(1), 111–115. https://doi.org/10.1016/j.jrp.2011.10.001

Pechorro, P., Castro, A., Hoyle, R. H., & Simões, M. R. (2018). The Brief Sensation-Seeking Scale: Latent Structure, Reliability, and Validity From a Sample of Youths At-Risk for Delinquency. *Journal of Forensic Psychology Research and Practice*, *18*(2), 99–113. https://doi.org/10.1080/24732850.2018.1435073
