# Supplementary material for: Positive and Negative Risk-Taking in Adolescence and Early Adulthood: A Citizen Science Study During the COVID-19 Pandemic
Source: Front Psychol. 2022 Jun 6;13:885692. doi: 10.3389/fpsyg.2022.885692 (PMC9207949; doi:10.3389/fpsyg.2022.885692)
Supplement: Supplementary file 2 [file Data_Sheet_2.PDF]

Supplementary Material S2
Calculation Example of High and Low Positive Risk-taking

Figure S2

Calculation of Positive Risk-taking to Peers versus Elderly (i.e., Non-Adherence to Social Distancing and Contract Restriction) with an Example of a High (Panel A) and Low (Panel B) Risk-taking Participant

A

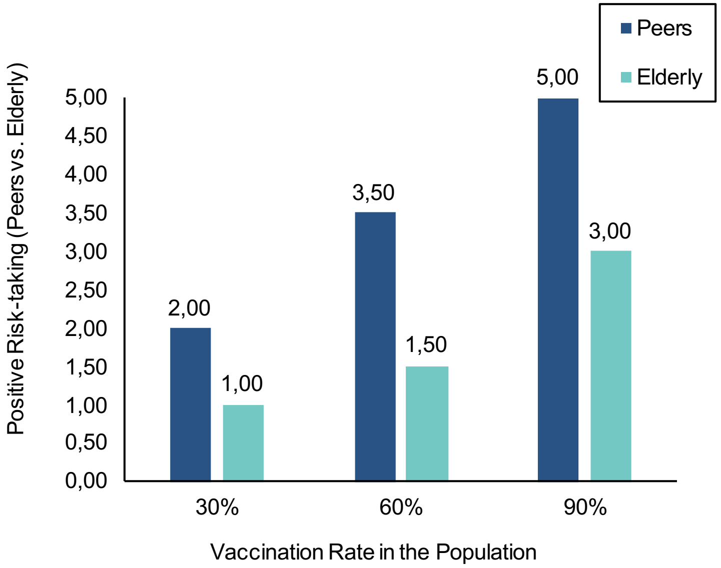

Mean peers:	3,50
Mean elderly:	1,83
**Mean difference:**	**1,67**

Mean 30%:	1,50
Mean 90%	4,00
**Mean increase:**	**2,50**

**Socially Accepted Risk-taking**	
1,67 * 2,50	**4,18**

B

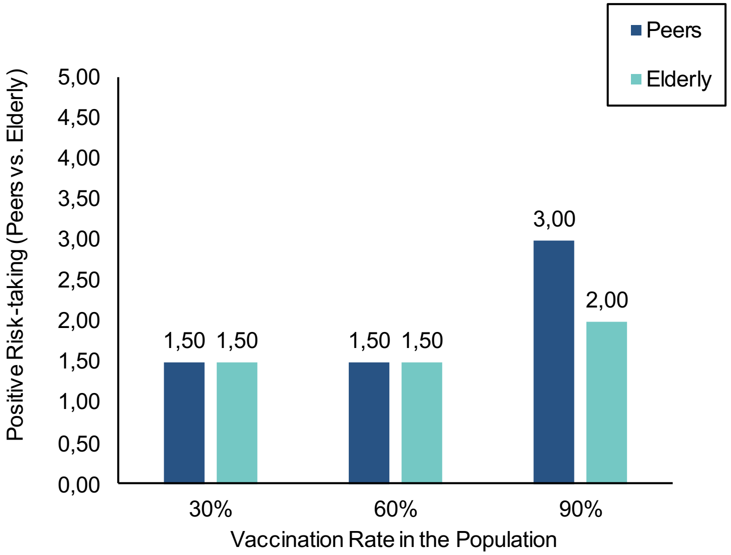

Mean peers:	2,00
Mean elderly:	1,67
**Mean difference:**	**0,33**

Mean 30%:	1,50
Mean 90%	2,50
**Mean increase:**	**1,00**

**Socially Accepted Risk-taking**	
0,33 * 1,00	**0,33**

Note. To prevent that participants with negative mean difference scores (mean peers < mean elderly) and negative mean increases (mean 30% > mean 90%) would receive positive risk-taking scores, negative mean increase scores were set to 1.00
