# Supplementary material for: Positive and Negative Risk-Taking in Adolescence and Early Adulthood: A Citizen Science Study During the COVID-19 Pandemic
Source: Front Psychol. 2022 Jun 6;13:885692. doi: 10.3389/fpsyg.2022.885692 (PMC9207949; doi:10.3389/fpsyg.2022.885692)
Supplement: Supplementary file 3 [file Data_Sheet_3.PDF]

Supplementary Material S3
Snowballing Data Collection Method: Timeline and Recruitment Text

In this supplementary document, we explain the snowballing data collection method and compare the data collection period with the timeline of Covid-19 restrictions. The data collection started on April 21st, 2021. The recruitment text (see Box C on the next page) was posted by the citizen scientists and researchers on several social media websites, and the citizen scientists circulated the text in several WhatsApp groups for adolescents and young adults. During the data collection period, social distancing (1,5 meter) and contact restrictions (i.e., a maximum number of visitors a day) were in place. However, some measures were relaxed (see Table A). Most participants answered the questionnaires during the first 10 days of the study start (see Figure B).

Table A. Overview of Covid-19 measures in the Netherlands during the data collection

**April 20st**	• Press conference, in which relaxation of measures from April 26th onwards were discussed by the prime minister.
**April 26th**	• Higher education is no longer (only) online
**April 28th**	• Outside terraces are open from 12:00 to 18:00.
	• Non-essential stores are open until 20.00.
	• No more evening lockdown (before, there was a strict evening lockdown from 22:00 to 4:30)
	• Citizens are allowed to receive a maximum of 2 visitors a day
**May 19th**	• Outside sport is allowed, gyms and swimming pools re-open (but no group lessons from age 18 onwards), with a maximum capacity of 30 people (and 1,5 meter distance)
	• Libraries and cultural institutions (i.e., music lessons) re-open, with a maximum capacity of 30 people per room (and 1,5 meter distance)

Figure B. Cumulative number of participants from the start of the study

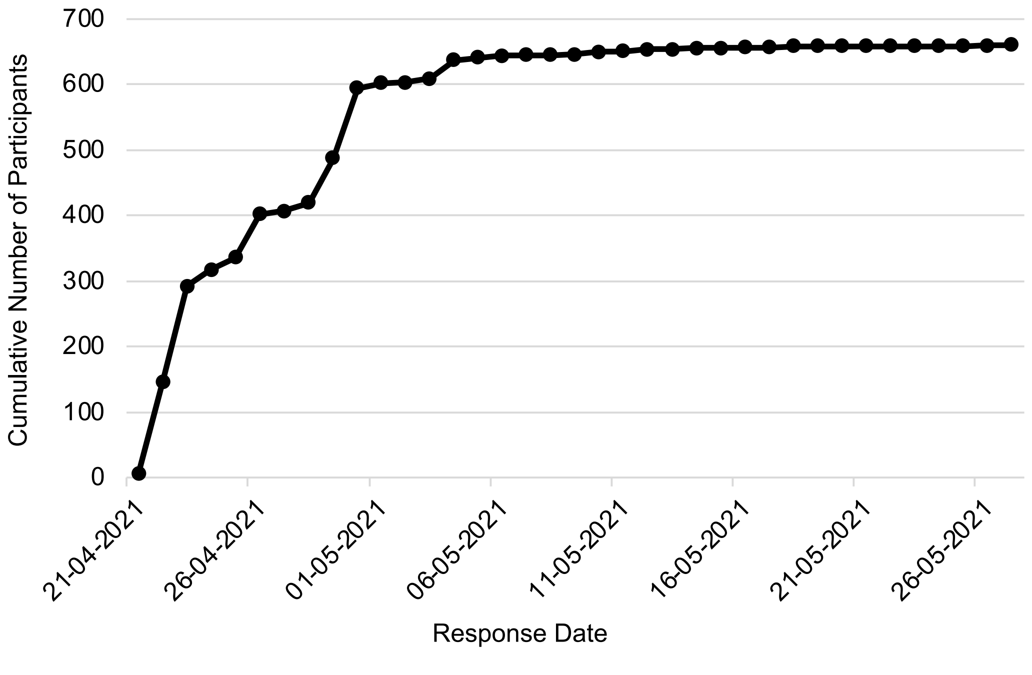

Box C. Recruitment Text made by the Citizen Scientists [translated from Dutch to English]

Soon, we can go to an outside terrace again! However, this does not mean that the Covid-19 crisis has ended. Measures, (self) testing and vaccinations will remain important. To ensure that the future decisions are adjusted to the needs of adolescents and students, the Lieve Mark students, together with YoungXperts scientists, map out the opinion of young people regarding 'the way out of the corona crisis'. The results of this survey will directly go to the RIVM and VWS.

So, fill in the questionnaire (this will take max 15 minutes) and forward this message to maximize support! As a thank you for participating, we will raffle four sets of AirPods Pro among the participants.

[link to information letter, content form and questionnaire]

An earlier questionnaire via a Whatsapp message has been completed more than 7,500 times and this report has been widely accepted by politicians. Time for another! Together we put the opinion of young people on the map!
