# Supplementary material for: Positive and Negative Risk-Taking in Adolescence and Early Adulthood: A Citizen Science Study During the COVID-19 Pandemic
Source: Front Psychol. 2022 Jun 6;13:885692. doi: 10.3389/fpsyg.2022.885692 (PMC9207949; doi:10.3389/fpsyg.2022.885692)
Supplement: Supplementary file 4 [file Data_Sheet_4.PDF]

Supplementary Material S4
Exploratory Subscale Analysis for the four Dimensions of Sensation Seeking

Method

The Brief Sensation-Seeking Scale measures four dimensions of sensation seeking (Hoyle et al., 2012); experience seeking (i.e., desire to seek experience for its own sake), boredom susceptibility (i.e., dislike of repetition of experience, being restlessness when things are unchanging), thrill and adventure seeking (i.e., desire to engage in sports or other activities involving elements of speed and danger), and disinhibition (i.e., loss of social inhibition). Each dimension includes two items. Although only two items per scale are not optimal, the developers of the measure (Hoyle et al., 2012) state that it is possible to combine pairs of items to form four content domains. In the current study, item-item correlations are .62 for experience seeking, .48 for boredom susceptibility, .60 for thrill and adventure seeking, and .64 for disinhibition (all significant at the .01 level). The between-domain correlations, and correlations with age and gender are reported in Table A.

Results

The results of regression analyses with risk-taking as dependent variable, gender and age as covariates and the four dimensions of sensation seeking as predictors can be found in Table B. Only the disinhibition subscale was a significant predictor of positive and negative risk-taking. Thus, participants with higher levels of disinhibition (e.g., "I would love to have new and exciting experiences, even if they are illegal") took more took more positive risks during Covid-19 (i.e., larger differences between non-adherence to social distancing in relation to peers versus elderly people, and high versus low vaccination rates), but also more negative Covid-19 related risks (i.e., they were more inclined to falsify test results or contract Covid-19 deliberately).

The results of regression analyses with societal contribution and life satisfaction as dependent variables, gender and age as covariates and the four dimensions of sensation seeking as predictors can be found in Table C. The subscale experience seeking was a significant positive predictor of societal needs, whereas the subscale thrill and adventure seeking was a significant positive predictor of societal opportunities. Thus, participants with higher levels of experience seeking ("I would like to explore strange places") reported a stronger need to contribute to society during Covid-19 (i.e., speak up for change, help society members). In contrast, participants with higher levels of thrill and adventure seeking (e.g., "I would like to try bungee jumping") perceived more opportunities to contribute to society (i.e., they felt more valuable to society during the Covid-19 crisis). Lastly, the subscale boredom susceptibility was a significant negative predictor of life satisfaction. Thus, participants who reported higher levels of boredom susceptibility (e.g., "I get restless when I spend much time at home") reported lower levels of life satisfaction during the Covid-19 crisis.

Interpretation/Discussion

Previous research has shown that the four domains of sensation seeking (i.e., experience seeking, boredom susceptibility, thrill and adventure seeking, and disinhibition). are differentially related to positive and negative (risk-taking) outcomes (Glicksohn, & Bozna, 2000; Glicksohn, Naor-Ziv, & Leshem, 2018; Glicksohn, & Rechtman, 2011). Specifically, it is reported that the subscale disinhibition relates to antisocial, negative forms of risk-taking, whereas the subscale thrill and adventure seeking relates to prosocial, positive forms of risk-

taking (Glicksohn, Naor-Ziv, & Leshem, 2018). The current study partly replicates these findings, although our results should be interpreted with caution, due to the use of the Brief Sensation-Seeking Scale with only two items per domain (Hoyle et al., 2021). In line with previous research, we found that the disinhibition dimension of sensation seeking was associated with negative (but also positive) Covid-19 related risk-taking, whereas the subscale thrill and adventure seeking was associated with the experienced opportunities for societal contribution. Future research should examine these potential differences between sensation seeking domains in depth, by incorporating the 40-item Sensation Seeking Scale (SSS-V; Zuckerman, Eysenck, & Eysenck, 1978).

Table A. Number of Participants, Descriptive Statistics and Correlations Among Dimensions of Sensation Seeking

	N	*M*	*SD*	1.	2.	3.	4.	5.
1. Age	660	22.91	3.14	-				
2. Gender[a]	651	0.70	0.46	.08*	-			
3. Experience seeking	614	3.76	1.06	.00	-.01	-		
4. Boredom susceptibility	628	3.70	1.02	-.13**	.04	.51**	-	
5. Thrill and adventure seeking	659	3.02	1.26	-.18**	-.13**	.34**	.34**	-
6. Disinhibition	617	2.97	1.20	-.15**	-.14**	.36**	.47**	.49**

Note. [a] Gender is coded as 0 (boy) 1 (girl); *indicates $p < 0.05$; ** indicates $p < 0.01$.

Table B. Coefficients for Stepwise Regression Analyses with Age, Gender (Step 1), and the four Dimensions of Sensation Seeking (Step 2) as Predictors of Negative Risk-taking and Positive Risk-taking

	Negative risk-taking[b]					Positive risk-taking[c]				
	b	*SE*	β	*p*	*p'*	*b*	*SE*	β	*p*	*p'*
Gender[a]	-0.05	0.06	-0.04	.384	>.999	0.07	0.09	0.03	.412	.824
Age	0.00	0.01	0.00	.999	>.999	-0.06	0.01	-0.17	<.001	<.001
Experience seeking	0.02	0.03	0.03	.526	>.999	-0.03	0.05	-0.03	.553	.824
Boredom susceptibility	0.02	0.03	0.03	.553	>.999	0.12	0.05	0.11	.023	.092
Thrill and adventure seeking	-0.01	0.03	-0.01	.771	>.999	-0.05	0.04	-0.06	.194	.582
Disinhibition	0.12	0.03	0.21	<.001	<.001	0.17	0.04	0.19	<.001	<.001

Note. p' is the Holm-Bonferroni adjusted *p*-value. [a] Gender is coded as 0 (boy) 1 (girl).
Change statistics of adding sensation seeking when age and gender are already in the regression:
[b] $\Delta R^2 = .05$, $\Delta F(4,598) = 7.98$, $p < .001$;
[c] $\Delta R^2 = .05$, $\Delta F(4,598) = 8.40$, $p < .001$;

Table C. Coefficients for Stepwise Regression Analyses with Age, Gender (Step 1), Risk-Taking (Step 2) and Sensation Seeking (Step 3) as Predictors of Societal Needs, Societal Opportunities, and Life Satisfaction

	Societal needs[b]					Societal opportunities[c]					Life satisfaction[d]				
	b	*SE*	β	*p*	*p'*	*b*	*SE*	β	*p*	*p'*	*b*	*SE*	β	*p*	*p'*
Gender[a]	0.26	0.10	0.11	.009	.045	0.25	0.11	0.10	.023	.115	-0.01	0.14	-0.00	.934	>.999
Age	-0.03	0.02	-0.08	.047	.188	0.02	0.02	0.04	.344	.752	0.04	0.02	0.08	.064	.320
Experience seeking	0.24	0.05	0.22	<.001	<.000	0.02	0.06	0.02	.696	.752	0.01	0.07	0.01	.923	>.999
Boredom susceptibility	0.05	0.06	0.04	.387	>.999	-0.07	0.06	-0.06	.225	.752	-0.31	0.08	-0.20	<.001	.001
Thrill and adventure seeking	0.01	0.04	0.01	.879	>.999	0.15	0.05	0.15	.002	.012	-0.03	0.06	-0.02	.684	>.999
Disinhibition	-0.00	0.05	-0.00	.952	>.999	-0.07	0.05	-0.07	.188	.752	-0.04	0.07	-0.03	.564	>.999

Note. p' is the Holm-Bonferroni adjusted *p*-value. [a] Gender is coded as 0 (boy) 1 (girl).
Change statistics of adding sensation seeking when age and gender are already in the regression:
[b] $\Delta R^2 = .06$, $\Delta F(4,596) = 9.63$, $p < .001$;
[c] $\Delta R^2 = .02$, $\Delta F(4,596) = 2.87$, $p = .023$;
[d] $\Delta R^2 = .06$, $\Delta F(4,592) = 7.16$, $p < .001$;
